# Cellular metabolism regulates the differentiation and function of T-cell subsets

**DOI:** 10.1038/s41423-024-01148-8

**Published:** 2024-04-02

**Authors:** Sicong Ma, Yanan Ming, Jingxia Wu, Guoliang Cui

**Affiliations:** Key Laboratory of Immune Response and Immunotherapy, Center for Advanced Interdisciplinary Science and Biomedicine of IHM, School of Basic Medical Sciences, Division of Life Sciences and Medicine, University of Science and Technology of China, Hefei, 230601 China

**Keywords:** Metabolism, Immunometabolism, T cell differentiation, CD4+ T cells, CD8+T cells, Tumour immunology, Adaptive immunity

## Abstract

T cells are an important component of adaptive immunity and protect the host from infectious diseases and cancers. However, uncontrolled T cell immunity may cause autoimmune disorders. In both situations, antigen-specific T cells undergo clonal expansion upon the engagement and activation of antigens. Cellular metabolism is reprogrammed to meet the increase in bioenergetic and biosynthetic demands associated with effector T cell expansion. Metabolites not only serve as building blocks or energy sources to fuel cell growth and expansion but also regulate a broad spectrum of cellular signals that instruct the differentiation of multiple T cell subsets. The realm of immunometabolism research is undergoing swift advancements. Encapsulating all the recent progress within this concise review in not possible. Instead, our objective is to provide a succinct introduction to this swiftly progressing research, concentrating on the metabolic intricacies of three pivotal nutrient classes—lipids, glucose, and amino acids—in T cells. We shed light on recent investigations elucidating the roles of these three groups of metabolites in mediating the metabolic and immune functions of T cells. Moreover, we delve into the prospect of “editing” metabolic pathways within T cells using pharmacological or genetic approaches, with the aim of synergizing this approach with existing immunotherapies and enhancing the efficacy of antitumor and antiinfection immune responses.

## Introduction

T cells, like other types of cells, engage in constant communication with their environment. The ability to interpret and respond appropriately to environmental signals, including cellular metabolic conditions, is crucial in determining the status of T cells and allowing them to adapt to their surroundings. Consequently, T cells must sense extracellular nutrients, maintain flexible intracellular metabolic pathways to align with nutrient availability, and support bioenergetics and biosynthesis for T cell survival, subset differentiation, and function. T cells not only passively adapt to their surroundings but also actively shape the environment by producing metabolites such as lactate. These metabolites influence the survival and function of neighboring cells within the same environment, thereby establishing bidirectional metabolic communication between T cells and their surroundings. T cell metabolism serves as a network that connects T cells and neighboring cells during complex immune responses in both normal and diseased settings. A comprehensive understanding of recent advancements in T cell metabolism is instrumental for identifying key hubs within this metabolic network and exploring potential metabolic drug targets for the development of the next generation of T cell-based immunotherapies.

A paradigm shift in immunometabolism emphasizes the fact that metabolites are not merely building blocks for biosynthesis or substrates for energy generation; they also directly or indirectly interact with major signaling hubs in T cells, thereby regulating the lineage differentiation of T cells. For instance, serine, in addition to being a crucial substrate for protein synthesis, also contributes to the biosynthesis of sphingolipids. Sphingolipids, in turn, modulate the activation of the mechanistic target of rapamycin complex (mTORC), thereby inhibiting ER stress-induced cell death [1]. The signaling-regulatory role of metabolites presents promising therapeutic opportunities for rewiring cellular metabolism and improving T cell-based therapies.

Lipids, glucose, and amino acids are extensively studied metabolites that underpin T cell survival, activation, and lineage differentiation [2–4]. In this review, we will use these three groups of metabolites as examples to explore major advancements in understanding how cellular metabolism regulates CD4+ and CD8 + T cell subset differentiation and their responses to invading pathogens, cancers, and self-antigens.

## Regulation of CD4 + T cell lineage commitment and function by lipid metabolism

### Fatty acids and CD4 + T cell differentiation

**Fig. 1:**
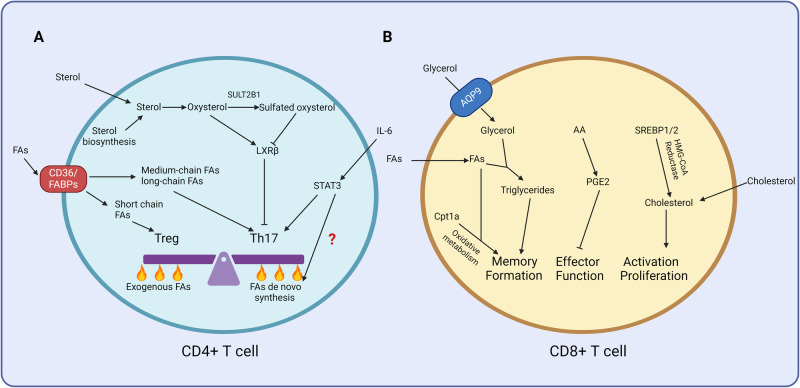
Regulation of T cell activation and subset differentiation and function by lipid metabolism. **A** The length of the fatty acid chain influences CD4 + T cell differentiation. Furthermore, Treg cells and Th17 cells employ distinct strategies to obtain fatty acids (i.e., importing exogenous fatty acids or synthesizing fatty acids). The IL-6-STAT3 signaling pathway is expected to play a key role in the decision-making between these two strategies. In addition, the sterol supply not only provides essential substrates for T cell clonal expansion but also modulates Th17 differentiation through the LXR pathway. **B** Fatty acid oxidation promotes memory CD8 + T cell formation. The AA-PGE2 pathway suppresses CD8 + T cell effector function. Endogenous cholesterol synthesis supports CD8 + T cell activation and proliferation. Supplementation with cholesterol rescues CD8 + T cell proliferation with defects in cholesterol synthesis. FAs fatty acids, LXR liver-X receptor, AQP9 aquaporin-9, AA arachidonic acid, PGE2 prostaglandin 2. Images were created with Biorender.com, with permission

Accumulating evidence suggests that how T cells acquire metabolites plays a pivotal role in determining cell differentiation outcomes, in addition to the impact of the metabolites themselves. For instance, interleukin-17-producing helper T (Th17) cells and regulatory T (Treg) cells employ distinct strategies for obtaining fatty acids (Fig. 1A). Compared with Treg cells, Th17 cells exhibit a greater reliance on the de novo synthesis of fatty acids. Th17 cells are particularly sensitive to the inhibition of endogenous acetyl-coenzyme A (acetyl-CoA) carboxylase 1-mediated fatty acid synthesis [5]. Inhibiting fatty acid synthesis not only significantly suppresses Th17 cell subset commitment but also promotes the differentiation of Treg cells.

The fact that Treg cells do not rely on cell-intrinsic fatty acid synthesis raises a crucial question: how do Tregs acquire the fatty acids necessary to support their bioenergetics and biosynthesis? Treg cells may enhance their dependence on extracellular fatty acids to fulfill the metabolic requirements for lipid synthesis. For instance, the inhibition of fatty acid binding proteins (FABPs) affects mitochondrial integrity, leading to the release of mitochondrial DNA and the initiation of cGAS/STING-dependent type I interferon signaling. This activation, in turn, promotes IL-10 production by Treg cells [6]. Genetic deficiency of *Cd36* diminishes the uptake of fatty acids by Treg cells, resulting in intratumoral Treg cell apoptosis and impeding tumor growth [7]. A notable advantage of this shift from biosynthesis to the import of fatty acids is a reduction in energy-consuming anabolism. However, this increased reliance on extracellular fatty acids also renders Treg cells susceptible to fluctuations in the availability of fatty acids in the environment. Consequently, a potential avenue for cancer therapeutics lies in inhibiting the uptake of fatty acids by Treg cells or restricting their access to extracellular fatty acids.

The fact that Th17 cells, as opposed to Treg cells, exhibit sensitivity to the inhibition of fatty acid de novo synthesis prompted an inquiry into the molecular mechanisms driving the preferential engagement of Th17 cells in de novo synthesis. The expression of FoxP3, which is a pivotal transcription factor for Treg cells [8–10], requires transforming growth factor (TGF)-β [11]. In contrast, Th17 cell polarization is promoted by TGFβ and interleukin (IL)-6 [12]. IL-6 activates signal transducer and activator of transcription 3 (STAT3). Hence, STAT3 could be dependent on fatty acid de novo synthesis in Th17 cells (Fig. 1A).

In addition to their distinct reliance on exogenous fatty acids, CD4+ Teff cells and Tregs are subject to different regulatory influences from fatty acids with varying carbon chain lengths. Fatty acids are categorized based on the number of aliphatic carbon tails: those with fewer than 6, 6–12, 13–21, and more than 21 are considered short-chain fatty acids, medium-chain fatty acids, long-chain fatty acids, and very long-chain fatty acids, respectively [13–15]. Short-chain fatty acids (e.g., propionate) play a role in promoting Treg differentiation and function. In contrast, long-chain fatty acids and medium-chain fatty acids such as lauric acid enhance Th1 cell and Th17 cell polarization at the expense of Treg cell formation [16]. Consequently, short-chain (propionate) and medium-chain (lauric acid) fatty acids have opposing effects on autoimmune encephalomyelitis (Fig. 1A).

The underlying mechanisms that determine the biological activity of fatty acids with different carbon chain lengths remain incompletely understood. Fatty acids serve as natural ligands for peroxisome proliferator-activated receptors (PPARs), which are transcription factors that regulate a wide array of genes involved in cell metabolism, differentiation, and function [17–25]. One plausible explanation is that fatty acids of various carbon chain lengths may regulate the conformational changes and transcriptional activities of PPARs in different or even opposing manners [26].

Another influential factor shaping the immune regulatory properties of fatty acids is their degree of saturation. Similar to carbon chain lengths, the double bonds in unsaturated fatty acid carbon chains induce conformational changes in PPARs and trigger PPAR-dependent intracellular signals in a manner different from the single bonds in saturated fatty acids [26]. In addition to their role in modulating signal transduction, fatty acids serve as a vital source of cellular energy that sustain cell growth, survival, and function through fatty acid β-oxidation. Notably, most fatty acids undergoing β-oxidation are unsaturated [27]. In other words, unsaturated fatty acids may function as a “preferred fuel” for cellular bioenergetics. This preference can alter the ratio between saturated and unsaturated fatty acids and indirectly influence PPAR ligand availability and transcriptional activity. Ultimately, the degree of saturation of fatty acids may determine their immune regulatory properties by influencing both the bioenergetics and cell signaling of T cells.

### Sterols and CD4 + T cell activation and polarization

Sterols are important components of cell membranes. The clonal expansion of CD4 + T cells is inherently linked to an increase in sterol demands, which can be met through the import of exogenous sterols or de novo synthesis (Fig. 1A). A key regulator of sterol transportation and synthesis is the liver-X receptor (LXR), which has two forms—LXRα and LXRβ. While LXRα is specifically expressed in adipose tissue, the liver, and macrophages, LXRβ is ubiquitously expressed [28]. Recent studies have suggested that LXRα and LXRβ are also expressed in T cells [28, 29]. Interestingly, LXRβ deficiency, rather than LXRα deficiency, increases CD4+ and CD8 + T cell proliferation. CD8 + T cells deficient in LXRβ produce significantly more effector cytokines (such as IFNγ) than do their wild-type counterparts during recall responses [30]. The mechanistic link lies in T cell activation, which upregulates the expression of SULT2B1, an enzyme responsible for adding a sulfate group to oxidized sterols. Unmodified oxidized sterols serve as natural ligands for LXRs, and SULT2B1-mediated sulfate modification leads to the inactivation of LXRs. Microarray analysis further revealed that T cell activation results in a decrease in the levels of the sterol transporters adenosine triphosphate (ATP)-binding cassette subfamily G member 1 and ATP-binding cassette subfamily A member 1, which are responsible for mediating the efflux of sterols or phospholipids to lipoproteins. Conversely, the expression of genes involved in sterol synthesis, such as 3-hydroxy-3-methylglutaryl-coenzyme A reductase (*Hmgcr*), and uptake, such as low-density lipoprotein receptor (*Ldlr*), is upregulated upon T cell receptor (TCR) ligation. This dynamic response suggests that upon TCR activation, T cells undergo rapid rewiring of their sterol metabolism, which is characterized by increased sterol synthesis and uptake to support membrane synthesis during T cell expansion.

Interestingly, the addition of mevalonate rescues the LXR-mediated suppression of T-cell proliferation. The mevalonate pathway generates five-carbon building blocks for synthesizing isoprenoids, which include cholesterols, vitamin K, and steroid hormones [31]. Additionally, isoprenylation, which is a crucial posttranslational protein modification, may occur on various proteins in mammalian cells, including T cells, macrophages, and hepatocytes [32–35]. Therefore, beyond accumulating sterols to support biosynthesis, the TCR ligation-driven mevalonate pathway may also induce the isoprenylation of specific proteins that regulate T cell activation. Notably, similar to T cells, macrophages have the flexibility to either import or synthesize sterols. Surprisingly, inhibiting the sterol biosynthetic pathway in macrophages spontaneously primes antiviral immune responses. These intriguing findings suggest that de novo sterol synthesis and import are not merely passively regulated by immune cell metabolic requirements. Instead, sterol metabolism actively influences macrophage-mediated immune responses through unknown mechanisms [36]. Whether and how de novo sterol synthesis and import differentially regulate the function and differentiation of CD4 + T cells has not been determined.

LXRs not only govern cell proliferation but also influence CD4 + T cell lineage commitment. For instance, prior studies have demonstrated that the activation of LXRs using synthetic agonists such as GW3965 and T0901317 suppresses Th17 cell differentiation in autoimmune diseases [29, 37, 38]. This inhibitory effect is mediated by sterol regulatory element-binding protein-1 (SREBP-1), which is a target gene of LXRs. SREBP-1 competes with the aryl hydrocarbon receptor (AhR) for binding to the *Il17a* locus, thereby suppressing AhR-driven *Il17a* transcription. These findings suggest that, beyond their role in regulating the biosynthesis of CD4 + T cell building blocks and supporting cell proliferation, the oxysterol receptor LXRs also play a pivotal role in modulating the differentiation of Teff cell subsets (Fig. 1A).

## Regulation of CD8 + T cell effector function and memory formation by lipid metabolism

### Fatty acid metabolism and memory CD8 + T cell longevity

One crucial aspect of our adaptive immune system is the establishment of immunological memory, which provides protection against repeated infections caused by pathogens. Long-lived immunity is a fundamental characteristic that is particularly evident in memory lymphocytes, such as memory CD8 + T cells, which acquire longevity as they develop in response to infection. During acute viral infections, antigen-specific CD8 + T cells undergo rapid and extensive clonal expansion and differentiate into Teff cells that secrete essential cytokines (including granzyme B, TNFα, and IFNγ) to effectively combat invading pathogens. Following pathogen clearance, most effector cells undergo apoptosis in the contraction phase, while a small population survives and persists as memory T cells [39].

Memory T cells can be categorized into three main subsets based on surface markers and migration patterns: central memory T cells, effector memory T cells, and noncirculating tissue-resident memory T cells. Circulating memory T cells, which are characterized by high expression of interleukin-7 (IL-7), Rα and IL-15Rβ, endure for extended periods, often years, and offer sustained and long-lasting protection against reinfection [40–42]. The longevity of memory T cells in various tissues relies on the cytokines IL-7 and IL-15, which play pivotal roles in promoting cell survival and self-renewal [43, 44]. Additional investigations have indicated that IL-7 and IL-15 promote the expression of the antiapoptotic protein Bcl-2, thereby facilitating memory T cell homeostatic turnover. This process is observed in lymphopenia-driven homeostatic proliferation, as well as in mice infected with various pathogens, such as vaccinia virus, *Listeria monocytogenes*, lymphocytic choriomeningitis virus (LCMV), or vesicular stomatitis virus [40, 41, 43–48].

In addition to upregulating Bcl-2 expression, recent studies have suggested that IL-7 and IL-15 also regulate memory T cell metabolism. IL-7 and, to a lesser extent, IL-15, induce the expression of the glycerol channel aquaporin-9 on memory CD8 + T cells after LCMV infection (Fig. 1B). Aquaporin-9 imports glycerol into memory CD8 + T cells, thereby contributing to the esterification of fatty acids and the formation of triglycerides [49]. Triglycerides, which are energy-rich molecules, serve as crucial energy reservoirs for memory T cells. The storage of triglycerides, which is facilitated by IL-7, allows memory T cells to maintain a stable and long-term energy supply, thereby reducing vulnerability to fluctuations in nutrient availability in the environment. Given that memory T cells can persist for extended periods, spanning years or even decades, IL-7-induced energy storage plays an indispensable role in ensuring the longevity of memory CD8 + T cells. However, to utilize the stored energy, triglycerides must be hydrolyzed into fatty acids, which are subsequently oxidized in the mitochondria to generate energy. The process of mobilizing triglycerides into nonesterified fatty acids requires lysosomal acid lipase in memory CD8^+^ T cells [50].

Additionally, IL-15 further augments the expression of carnitine palmitoyltransferase 1a (CPT1a), thus facilitating the transport of fatty acids into the mitochondria. This enhancement promotes oxidative phosphorylation and the formation of memory T cells, thereby contributing to their metabolic activity [51]. Overall, IL-7 and IL-15 collaborate to regulate cell-intrinsic lipogenesis and lipolysis to ensure stable cellular energetics and support the enduring persistence of memory CD8 + T cells. An intriguing question arising from this model is why memory T cells participate in a seemingly futile cycle of synthesizing and subsequently breaking down triglycerides. One plausible explanation is that triglyceride synthesis serves to secure energy deposits, thus mitigating the risk of passive dependence on environmental nutrients, as discussed earlier. In support of this notion, retroviral overexpression of CPT1a has been shown to enhance memory CD8 + T cell development [51]. In contrast, genetic deficiency of *Cpt1a* did not significantly impact the formation of memory CD8 + T cells [52]. These findings suggest that gain-of-function and loss-of-function CPT1a have distinct impacts on memory CD8 + T cell differentiation (Fig. 1B).

Similar to the circulating memory T cells described earlier, noncirculating tissue-resident memory T cells also rely on fatty acid metabolism for long-term maintenance. Tissue-resident memory T cells express high levels of FABP4 and FABP5 [53]. Deficiency of these two genes reduces the uptake of extracellular fatty acids, impairs fatty acid β-oxidation and mitochondrial oxidative metabolism, and adversely affects the long-term survival of skin tissue-resident memory T cells following viral infection [53]. In addition to FABP4 and FABP5, various other members of the FABP protein family are expressed in tissue-resident memory T cells, and this expression is tissue-specific [54]. For instance, gut tissue-resident memory T cells express FABP1, FABP2, and FABP6, while liver tissue-resident memory T cells express FABP1 and FABP4. Moreover, T cells acquire tissue-specific expression patterns of FABPs upon transfer to new tissue, indicating that tissue-specific factors dictate the expression profiles of FABPs. This result underscores the plasticity of fatty acid metabolism in tissue-resident memory T cells by highlighting their ability to adapt to tissue-specific environments [54]. Tumor-infiltrating T cells express markers indicative of tissue-resident memory cells [55]. Inhibition of the immune checkpoint molecule PD-L1 enhances the expression of FABP proteins, facilitates lipid uptake by tissue-resident memory cells, and improves the survival of these cells in patient-derived xenograft mouse models [56].

The expression of Bcl-2 or Ki-67 is positively correlated with the quantity of neutral lipids, such as triglycerides, in memory CD8 + T cells [49], suggesting that memory T cells undergoing homeostatic turnover and self-renewal have much higher levels of triglycerides than their undivided counterparts. These results suggest a close association between triglycerides and cell proliferation, potentially indicating a that memory T cells have to meet certain requirements to enter cell cycle progression. Considering the shared self-renewal and differentiation potential of stem cells and memory T cells, lipid metabolism must be important in regulating the dynamic behavior of memory T cells [57], and the findings described above using the antiviral memory CD8 + T cell model may also apply to stem cell homeostasis [58]. IL-7-mediated lipogenesis is specific to memory T cells, not naïve T cells, despite both subsets expressing high levels of IL-7Rα. One plausible explanation is that IL-7 alone may not be sufficient to activate the machinery necessary for glycerol uptake and fatty acid esterification. Additionally, yet-to-be-identified signals beyond IL-7 are also implicated in triglyceride synthesis in memory CD8 + T cells. Furthermore, distinctions in fatty acid-triglyceride metabolic strategies between naïve and memory CD8 + T cells underscore a metabolic dichotomy and distinct nutrient requirements for these two CD8 + T cell subsets. This dichotomy likely facilitates the coexistence of memory and naïve CD8 + T cells, thus preventing potential competition for the same pool of nutrients.

A population of PD-1+Tcf1 + CD8 + T cells, known as “stem-like T cells” or “progenitor-exhausted T cells”, display memory T-cell-like properties in tumors and chronic infections [59–61]. The metabolism of these “stem-like T cells” is poorly understood. PD-1 expression increases fatty acid oxidation at the expense of glycolysis [62, 63]. The survival of this subset of “stem-like T cells” is inhibited by oxidized low-density lipoprotein (oxLDL) metabolism. Genetic deletion of *Cd36* (encoding the oxLDL transportation protein CD36) increases the percentage of these “stem-like T cells” among tumor antigen-specific CD8 + T cells [64]. An important question in this field is which signaling pathways regulate the lipid metabolism of these “stem-like T cells”. Answering this question will offer new opportunities to enhance the metabolic fitness of these T cells. A recent study showed that pharmaceutical inhibition of MEK1/2 promotes fatty acid oxidation and confers a stem cell-like memory phenotype to CD8 + T cells [65]. Additional studies are required to further explore the signaling pathways that are involved in regulating lipid metabolism in these “stem-like T cells”.

### Prostaglandins and antiviral CD8 + T cells during chronic infection

Arachidonic acid can be metabolized into various groups of bioactive lipids, including prostaglandins (PGEs) [66–70]. Strategies involving pharmacological or genetic inhibition of PGE2 production and signaling, either alone or in combination with PD-1 blockade, have been shown to enhance CD8 + T cell effector function during chronic LCMV infection [71]. Using an influenza viral infection mouse model, Coulombe et al. showed that PGE2 signaling suppresses effector CD8 + T cell formation by inhibiting macrophage-produced type I interferon and antigen presentation [72]. In the context of breast cancer mouse models, fibroblasts within lung metastases express cyclooxygenase 2 and produce PGE2. These PGE2-producing fibroblasts induce an immunosuppressive phenotype in dendritic cells and monocytes. Consequently, “fibroblast-educated” myeloid cells exhibit a reduced capacity to prime T cells. A deficiency in *Ptgs2* (encoding cyclooxygenase 2) in fibroblasts increases T cell activation and enhances the antimetastatic activity of immunotherapies [73]. These studies suggest that PGE2 suppresses antiviral CD8^+^ T cell function in both CD8^+^ T cell-intrinsic and -extrinsic manners (Fig. 1B).

### Sterols and effector CD8 + T cell function

Cholesterol plays a pivotal role in modulating the function and expansion of CD8 + T cells. The enzyme that acts as the rate-limiting step in cholesterol biosynthesis is 3-hydroxy-3-methylglutaryl-coenzyme A (HMG-CoA) reductase [74–80]. Pharmacological inhibition of HMG-CoA reductase using statins suppresses mitogen-induced T lymphocyte expansion. This result suggests that sterol synthesis plays a crucial role in T cell activation and proliferation [81]. Interestingly, the expression level of HMG-CoA reductase is dramatically increased upon T cell activation [82] This finding aligns with another insightful study that showed that TCR engagement is linked to fundamental metabolic reprogramming [3]. HMG-CoA reductase is regulated by the transcription factors SREBP-1 and SREBP-2 [83–86] (Fig. 1B). The transcriptional activities of SREBPs are controlled at the posttranslational level. Transcriptionally inactive SREBP proteins are retained in the membrane of the endoplasmic reticulum. When intracellular sterol levels become limited, SREBP cleavage-activating protein (SCAP) escorts SREBPs to the Golgi apparatus. Here, premature SREBPs undergo cleavage and release active fragments that translocate into the nucleus to transactivate their target genes [87–90]. Previous studies utilizing SREBP-1 and SREBP-2 single-deficient mice suggest a potential compensatory mechanism in which the loss of one isoform may compensate for the loss of the other isoform [91, 92]. The transcriptional activities of both SREBP-1 and SREBP-2 are suppressed in *Scap* knockout mice [82]. Moreover, *Scap* deficiency does not impact T cell homeostasis. The immunosuppressive function and stability of intratumoral Treg cells are supported by Scap and SREBP-FASN (fatty acid synthase) axis activity. Genetic deletion of Scap in Treg cells delays tumor growth and enhances the response of CD8 + T cells to anti-PD1 immunotherapy [93]. However, the proliferation and antiviral responses of *Scap* knockout CD8 + T cells are significantly impaired, accompanied by reduced nutrient uptake, glycolysis, and mitochondrial oxidative phosphorylation. In addition to undergoing cell-intrinsic biosynthesis, CD8 + T cells also acquire sterols from the environment. Notably, supplementation with cholesterol in vitro corrects *Scap* deficiency-associated defects in cell proliferation and metabolism. These studies suggest that TCR activation directs SREBP-dependent reprogramming of sterol metabolism. The fact that antigen persistence desensitizes the TCR signals in CD8 + T cells highlights the complexity of T cell responses [94]. Further investigating how SCAP and SREBP regulate CD8 + T cell effector functions, particularly in the context of chronic infections or cancer development, would be interesting.

## Glucose metabolism in CD4 + T cells

### Glucose metabolism and CD4+ effector T cell lineage activation and commitment

**Fig. 2:**
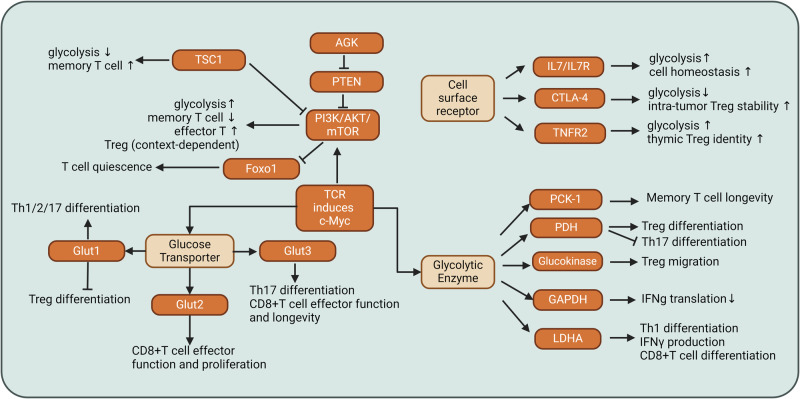
The glucose metabolism network regulates CD4+ and CD8 + T cell subset differentiation and function. The signaling network, which is composed of glucose transporters, glycolytic enzymes, and glycolysis-related intracellular signaling hubs and surface receptors, modulates T cell differentiation and function. These transporters, enzymes, signaling hubs, and surface receptors, many of which are regulated by TCR activation, not only support T cell glycolysis and bioenergetics but also regulate signal transduction, the epigenetic landscape, and T cell subset differentiation. CTLA-4 cytotoxic T-lymphocyte-associated protein 4, TSC1 tuberous sclerosis complex 1, PTEN phosphatase and tensin homolog. Images were created with Biorender.com, with permission

Upon T-cell receptor (TCR) activation and exposure to lineage-specific cytokines, naïve CD4 + T cells undergo differentiation into various lineages [95–97]. The differentiation process is intricately linked to the regulation of glycolysis rates. CD4 + T cell activation triggers upregulated expression of several glucose transporters (Glut), including Glut1, Glut3, Glut6, and Glut8 [98]. TCR stimulation and IL-7 promote glucose uptake and aerobic glycolysis [3, 98, 99]. The expression levels of glucose transporters, glycolytic enzymes, and transcription factors related to glycolysis collectively influence the differentiation of T cell subsets (Fig. 2).

A deficiency in *Slc2a1* (encoding the Glut1 protein) reduces the number of thymocytes, inhibits the expansion of effector CD4 + T cells, and diminishes cytokine production across various CD4 + T cell subsets, including interferon-gamma (IFNγ), interleukin-4 (IL-4), and interleukin-17 (IL-17). Notably, Glut1 is dispensable for the lineage commitment of CD4+ Treg cells. The proliferation and production of granzyme B are not dependent on Glut1 [98] (Fig. 2). The cell surface expression levels of Glut1 are greater in Th1, Th2, and Th17 cells than in Treg cells [100, 101]. Consistent with Glut1 expression patterns, Th1, Th2, and Th17 cells exhibit increased rates of glycolysis, while Treg cells exhibit increased rates of fatty acid oxidation. Pharmacological activation of AMP-activated protein kinase (AMPK) inhibits Glut1 expression, thereby promoting fatty acid oxidation and increasing FoxP3 expression, which is a lineage marker for Treg cells [101]. Inhibiting glycolysis through pharmacological approaches, such as the use of 2-deoxyglucose and rapamycin, or genetic approaches, such as *Hif1a* deficiency (encoding hypoxia-inducible factor 1α), favors Treg cell formation at the expense of Th17 cells [102]. CG-5, which is a structurally optimized derivative of the glucose uptake inhibitor CG-12 [103, 104], inhibits glucose uptake, suppresses Th1 and Th17 cell differentiation, and promotes Treg cell induction. Furthermore, CG-5 has been shown to ameliorate lupus in mouse models [105].

CD4 + T cells upregulate the expression of Glut3 upon TCR activation [106]. IL-12 and IL-6, which promote the differentiation of Th1 cells and Th17 cells, respectively, further increase the mRNA levels of *Slc2a3* (which encodes the Glut3 protein). *Slc2a3* deficiency inhibits Th17 cell differentiation and increases interferon-gamma (IFNγ) production by Th1 cells. In mouse models of autoimmune colitis and encephalomyelitis, Th17 cells rely on Glut3 to induce inflammation. Glut3 is indispensable for glucose uptake, mitochondrial glucose oxidation, and the subsequent production of acetyl-CoA. Acetyl-CoA plays a pivotal role in regulating the chromatic accessibility of loci associated with inflammatory genes, including *Il17a* [106]. Glut3 expression has also been detected in Treg cells [107]. A side-by-side comparison revealed that the expression levels of Glut3 in Treg cells surpassed those in Teff cells. Further investigations are warranted to explore the role of Glut3 in Treg cell lineage commitment.

In addition to glucose transporters, glycolytic enzymes also regulate the differentiation and effector function of CD4 + T cells. TCR stimulation induces the expression of lactate dehydrogenase A (LDHA) [108]. Deficiency in *Ldha* reduces the availability of acetyl coenzyme A. Acetyl coenzyme A is required for the recruitment of H3K9 acetylation (H3K9Ac) to the *Ifng* promoter and noncoding sequence located −22 kb from the transcription start site of the *Ifng* gene (CNS22) enhancer region [108, 109]. Therefore, LDHA promotes IFNγ expression through an epigenetic program. This finding is consistent with studies showing that histone acetylation is contingent upon the glucose metabolism-related enzyme ATP-citrate lyase [110] (Fig. 2).

Pyruvate dehydrogenase (PDH) promotes the transportation of pyruvate into mitochondria, where it is converted to acetyl-CoA [111–114]. Acetyl-CoA, which is a metabolic substrate, enters the tricarboxylic acid (TCA) cycle to help maintain mitochondrial oxidation. PDH-dependent generation of acetyl-CoA is crucial for H3K27 histone acetylation and remodeling of the genetic landscape associated with CD4 + T cell activation [115]. PDH function can be modulated by pyruvate dehydrogenase kinase 1 (PDHK1), which phosphorylates PDH, thereby inhibiting its activity [116]. This phosphorylation impedes the transportation of pyruvate into mitochondria, leading to increased conversion of pyruvate to lactate [117]. Th17 cells express higher levels of PDHK1 than Treg cells, accompanied by elevated glycolytic rates in Th17 cells relative to Treg cells (Fig. 2). Pharmacological inhibition of PDHK1 using dichloroacetate (DCA) or knockdown of PDHK1 promotes Treg cell differentiation at the expense of Th17 cells [118–121]. Suppression of PDHK1 effectively inhibits the development of experimental autoimmune encephalomyelitis (EAE). PDHK1 inhibition not only suppresses glycolysis but also promotes mitochondrial oxidation metabolism and reactive oxygen species (ROS) production. The use of N-acetyl cysteine (NAC), which is known for its ability to reduce ROS levels, rescues Th17 cell formation from dichloroacetate (DCA)-induced suppression [118].

Glyceraldehyde 3-phosphate dehydrogenase (GAPDH) is a glycolytic enzyme that catalyzes the 6^th^ step of glycolysis, in which glyceraldehyde 3-phosphate is converted to D-glycerate 1,3-bisphosphate. The catalytic cysteine residue of GAPDH is succinated by dimethyl fumarate [122]. Therefore, dimethyl fumarate inactivates GAPDH and inhibits aerobic glycolysis in activated CD4 + T cells, which explains its immunomodulatory role in treating autoimmune diseases, such as multiple sclerosis and psoriasis. In addition to its well-established role in glucose breakdown, GAPDH has multiple nonmetabolic functions. GAPDH is an mRNA-binding protein that regulates mRNA nuclear export [123] and influences mRNA stability and translation [124–129]. GAPDH inhibits the translation of IFNγ in CD4 + T cells by binding to the 3’ untranslated region (UTR) of *Ifng* mRNA [130] (Fig. 2). The availability of glucose influences the allocation of the GAPDH protein between glycolysis and mRNA binding events, thereby intricately regulating the translation of target mRNAs.

In addition to glucose transporters and glycolytic enzymes, transcription factors play a key role in guiding the activation and lineage commitment of CD4 + T cell subsets. Hypoxia-inducible factor 1 α subunit (HIF1α) and cellular myelocytomatosis oncogene (c-Myc) are among the top candidate transcription factors predicted to regulate T cell activation-associated metabolic reprogramming. Oxygen limitation inhibits the expression of genes encoding mitochondrial respiration-related proteins and promotes the expression of proteins associated with glucose transportation and glycolysis [131, 132]. HIF1α is required for embryonic stem cell expression of glucose transporters and glycolytic enzymes [133]. Genes encoding several glycolytic enzymes, such as phosphoglycerate kinase 1, enolase, aldolase A, LDHA, and phosphofructokinase, have been proposed to be direct targets of HIF1α [134–138]. Although the expression of HIF1α is rapidly induced by T cell activation, HIF1α is dispensable for T cell activation-induced glycolysis in T cells [3]. Th17 cells express higher levels of HIF1α than Th1, Th2, or Treg cells. High expression levels of HIF1α are dependent on the mechanistic target of rapamycin (mTOR). Genetic ablation of *Hif1a* inhibits Th17 cell differentiation while enhancing Treg cell lineage commitment [139]. HIF1α plays a dual role in T cell differentiation by binding to FoxP3, promoting its proteasomal degradation and thereby inhibiting Treg cell development [140]. Conversely, HIF1α recruits RORγt and p300 to the *Il17a* promoter, thereby transactivating *Il17a* expression and promoting Th17 cell differentiation.

c-Myc has been demonstrated to play a crucial role in promoting the expression of genes associated with glycolysis, including Glut1, Glut2, Glut3, Glut4, hexokinase, phosphoglucose isomerase, phosphofructokinase, aldolase A, triose phosphate isomerase, glyceraldehyde-3-phosphate dehydrogenase, phosphoglycerate kinase 1, phosphoglycerate mutase, enolase, pyruvate kinase, and LDHA [141] (Fig. 2). c-Myc not only promotes the expression of LDHA to convert pyruvate to lactate, thereby promoting the extramitochondrial metabolism of pyruvate, but also increased the expression of mitochondrial transcription factor A and increases mitochondrial mass [142, 143]. T cell activation induces the expression of c-Myc, and c-Myc is required for T cells to rapidly engage in glycolysis [3]. The expression of c-Myc in T cells is positively regulated by the extracellular signal-regulated kinase (ERK), AKT (AKR mice with thymoma), and mTOR signaling pathways. Inhibition of these three pathways has been shown to reduce the expression of c-Myc in T cells [3]. All three pathways are known regulators of T cell metabolism [144–146].

### Glucose metabolism, Treg cell differentiation, and suppression

Treg and Teff cells have distinct glucose metabolic patterns. Treg cells express much lower levels of Glut1 and have lower glycolytic rates than Teff cells [101]. Glut1 is dispensable for Treg cell formation both in vitro and in vivo [100]. Moreover, Treg cells employ autophagy to suppress glycolysis and maintain lineage stability [147]. The immune checkpoints CTLA-4 and PD-1, which are highly expressed in Treg cells, suppress glycolysis [145]. CTLA-4 blockade increases glucose uptake by Treg cells, promotes IFNγ production, and destabilizes intratumor Treg cells [148] (Fig. 2).

AMPK is phosphorylated at higher levels in Treg cells than in Teff cells, indicating increased AMPK activity in Treg cells [101, 149–151]. Metformin, a compound known to activate AMPK [152], decreases Glut1 expression levels and promotes Treg cell formation in asthma [101]. Treg cells express lower levels of HIF1α than Th17 cells. Rapamycin, an inhibitor of mTOR, reduces the expression of HIF1α, blocks glycolysis, and promotes Treg cell differentiation [138, 139]. Hyperactive glycolysis represses Treg cell suppressive capacity and FoxP3 expression. For example, Treg cell-specific deletion of phosphatase and tensin homolog (PTEN) causes the hyperactivation of glycolysis and reduces FoxP3 stability, thereby impairing the suppression of Th1 cell and follicular helper T (Tfh) cell immune responses [153]. Furthermore, toll-like receptor (TLR)-mediated signals markedly increase Glut1 expression and glycolytic rates, which is detrimental to the stability and suppressive capacity of Treg cells [154]. Additionally, autophagy deficiency in Treg cells leads to an increase in glycolysis, which impairs the survival and lineage stability of Treg cells [147] (Fig. 2).

Although mTOR deficiency does not affect initial T cell activation, it impairs the lineage commitment of Th1, Th2, and Th17 cells. In contrast, mTOR deficiency leads to hyperactivation of the TGFβ-Smad3 pathway and promotes Treg cell formation [155]. Rapamycin expands Treg cells [156], suggesting that mTORC1 promotes Treg cell generation. mTOR inhibition promotes fatty acid oxidation and mitochondrial respiration, which are essential for Treg cell lineage commitment. This model has been challenged by experimental evidence showing higher levels of mTORC1 activation in Treg cells than in non-Treg T cells [157, 158]. These studies suggest that mTOR plays a more complicated role than originally proposed and may function in a context-dependent manner to drive T cell subset differentiation under various pathological or physiological conditions. Similarly, although excessive glucose catabolism is undesirable for Treg cell identity maintenance, Treg cells still require glycolysis to maintain their suppressive function. For example, tumor necrosis factor receptor 2 (TNFR2)-mediated costimulatory signals promote a glycolytic switch in human thymus-derived Treg cells [159] (Fig. 2). Notably, these glycolytic Treg cells are metabolically distinct from glycolytic Teff cells in that these Treg cells prefer to fuel glucose-derived pyruvate into the TCA cycle, in contrast with Teff cells, in which pyruvate is preferentially metabolized into lactate. TNFR2-driven glycolysis is required to maintain the identity and suppressive function of Treg cells [159]. Furthermore, Treg cells require the phosphoinositide 3-kinase (PI3K)-mTORC2 pathway to induce expression of the glycolytic enzyme glucokinase [160]. Glucokinase associates with actin and promotes cytoskeletal rearrangements, followed by migration to inflammatory tissue. Human Treg cells with increased glucokinase activity display increased migratory activity [160]. Therefore, the roles of mTOR and glycolysis in regulating Treg cell differentiation, function, and migration are context dependent.

Treg cells are more adaptable to high-lactate and low-glucose microenvironments in tumors than Teff cells. FoxP3, the lineage regulator of Treg cells, suppresses the anti-CD3/28 stimulation-induced phosphorylation and activation of Akt and reduces the expression of Glut1 in human CD4 + T cells [161]. SC79, which activates Akt [162], promotes the phosphorylation and activation of Akt and promotes Glut1 surface expression in Treg cells [161]. The FoxP3 protein is directly bound to the TATA box region of the *Myc* locus. FoxP3 inhibits the expression of c-Myc, reduces the expression of glycolysis-related genes, and promotes the mitochondrial oxidation of NADH, leading to an increase in the NAD+:NADH ratio [163]. NAD+ is required for the conversion of lactate into pyruvate. An increase in the NAD+:NADH ratio allows Treg cells to use lactate as a substrate to generate pyruvate for subsequent mitochondrial bioenergetics in a high-lactate and low-glucose microenvironment, such as the tumor microenvironment (TME) [163]. Abrogation of lactate transporters impairs the suppressive function of Treg cells, thus unleashing antitumor immune responses [164]. In highly glycolytic tumors, Treg cells take up lactate through monocarboxylate transporter 1. Lactate promotes the nuclear translocation of nuclear factor of activated T cells (NFAT)-1, which increases the expression of PD-1 on Treg cells [165]. PD-1 blockade promotes the expression of CTLA-4, ICOS, and GITR on Treg cells and reduces therapeutic efficacy.

## Glucose metabolism and CD8 + T cell activation and differentiation

Similar to CD4 + T cells, CD8 + T cell activation and differentiation are regulated by glycolysis-related glucose transporters, enzymes, transcription factors, mTOR complexes, cytokines, adipokines, and small-molecule metabolites. In addition to these similarities, there are also marked differences in the role of glycolysis regulation in CD4 + T cells and CD8 + T cells.

CD8 + T cells do not depend on Glut1 to proliferate or produce granzyme B [98]. CD8 + T cells require Glut2 for optimal proliferation and effector cytokine production. A deficiency in *Slc2a2* (encoding Glut2) affects CD8 + T-cell-mediated anti-allograft immune reactions [166]. Glut2 “senses” the availability of glucose in the microenvironment, and its expression is regulated by the availability of glucose and the concentration of oxygen. HIF1α, which is induced under low-oxygen conditions, inhibits Glut2 expression by CD8 + T cells [166]. Galectin-9, which is the ligand of Tim-3 [167] and is induced by HIF1α [168], inhibits the expression of Glut2. Stomatin, which is located at the immunological synapse during T cell activation and promotes Teff cell responses [169], enhances the surface expression of Glut2. The single nucleotide polymorphism rs5400 in *SLC2A2* in human CD8 + T cells reduces glucose uptake and inhibits CD8^+^ T cell proliferation [98]. In memory CD8 + T cells, the expression of Glut1 and Glut3 increases, and the glycolytic rate increases when the oxygen concentration decreases from 20% to 3% [170]. Overexpression of Glut3 in murine CD8 + T cells increases glucose uptake and energy storage, improves the metabolic fitness of mitochondria, and tightly regulates B16 melanoma tumor growth [171]. CD28 promotes the surface expression of Glut1 and glycolysis [145]. CD28 costimulation also increases the expression of Glut3, promotes glycolysis, and enhances CD8 + T cell function in renal cell carcinoma [172]. Furthermore, CD28 engagement increases the expression of Cpt1a and promotes mitochondrial fatty acid oxidation in CD8 + T cells [173].

Hexokinase 2 catalyzes a rate-limiting step in the glycolytic pathway. When cellular ROS reach threshold levels, HK2 is degraded through autophagy. NF-κB-inducing kinase (NIK, encoded by the gene *Map3k14*) is a mediator of noncanonical NF-κB activation [174]. NIK promotes the expression of glucose-6-phosphate dehydrogenase, which is required for the generation of NADPH [175, 176]. *Map3k14* deficiency disrupts NADPH production, induces the accumulation of ROS in cells, and eventually leads to the autophagic degradation of hexokinase 2. Moreover, *Map3k14* deficiency does not influence T cell development in the thymus [177]: and *Map3k14*-deficient CD8 + T cells exhibit decreased glycolytic rates and impaired antitumor responses [178].

As mentioned above, PDHK1 regulates T-cell subset differentiation. PDHK1 is expressed at higher levels in Th17 cells than in Treg cells and is accompanied by higher rates of glycolysis in Th17 cells than in Treg cells [118]. PDHK1 promotes Th17 cell differentiation and inhibits Treg cell lineage commitment [118]. In CD8 + T cells, TCR stimulation rapidly increases the phosphorylation of tyrosine residues in PDHK1 [117]. PDHK1 is phosphorylated in cancer cells, and this protein modification promotes the role of PDHK1 in glycolysis [179]. Acutely activated T cells require PDHK1 to synthesize the cytokines IFNγ, TNFα, and IL-2, but PDHK1 is not required for T cell-mediated cytotoxicity. LDH binds to the AU-rich element (ARE) in the 3′UTRs of *Ifng*, *Tnf*, and *Il2* mRNAs and represses the translation of the three proteins. PDHK1 inhibits PDK, thereby diverting LDH to exert its glycolytic function and alleviating LDH-mediated repression of *Ifng*, *Tnf*, and *Il2* mRNA translation [117].

High LDHA expression in tumor cells is characterized by lactate accumulation. Lactate suppresses the expression of NFAT in CD8 + T cells and IFN-γ generation [180]. Notably, lactate has been shown to increase the nuclear translocation of NFAT1 in Treg cells [165], suggesting that lactate may regulate NFAT protein expression and subcellular localization in a context-dependent manner. Similar to CD4 + T cells, in which LDHA is induced by TCR stimulation [108], antigen-specific CD8 + T cells also upregulate the expression of LDHA upon activation [181]. PI3K signaling is required for LDHA induction in CD8 + T cells. LDHA, in turn, promotes PI3K signal transduction and the phosphorylation of downstream signaling pathway components, including Akt and FoxO1. LDHA is required to maintain the NAD+:NADH ratio and cellular redox balance and to sustain the production of ATP [181]. However, LDH has been shown to inhibit T cell stemness. Inhibition of LDH synergizes with IL-21 to promote T cell stemness and durable antitumor CD8 + T cell responses [182] (Fig. 2).

Compared with naïve CD8 + T cells, memory CD8 + T cells are more efficient at importing glucose and engaging in glycolysis when subjected to antigen challenge [183]. In the early phase after antigen challenge and before cell proliferation starts, the activity of the key glycolytic enzyme GAPDH is greater in memory CD8 + T cells than in naïve CD8 + T cells. This antigen challenge-induced early glycolysis is further sustained by the CD28/Akt signaling and mTORC2 pathways. Inhibiting glycolysis reduces the production of IFNγ by memory CD8 + T cells [183], consistent with the findings of another study showing that the nonglycolytic enzyme GAPDH inhibits the translation of IFNγ mRNA in CD4 + T cells [130]. GAPDH is not only a glycolytic enzyme but also an mRNA-binding protein. GAPDH regulates mRNA nuclear export, stability, and translation [123–129]. Glucose availability determines the distribution of the GAPDH protein between glycolytic and nonglycolytic events, such as target mRNA translation.

CD8+ memory T cells express high levels of cytosolic phosphoenolpyruvate carboxykinase (Pck1). Pck1 promoted glycogenesis via gluconeogenesis in CD8 + T cells (Fig. 2). The glycogen synthesized in CD8 + T cells is metabolized to generate glucose-6-phosphate. Glucose-6-phosphate is then channeled to the pentose phosphate pathway to produce NADPH. Memory T cells require NADPH to maintain high levels of glutathione. This Pck1-glycogen-PPP-NADPH-glutathione metabolic pathway promotes memory CD8 + T cell formation [184].

NFATc1, but not NFATc2, is required for CD8 + T cells to engage in the T cell activation-induced metabolic switch to glycolysis [185]. The addition of IL-2 partially restored glycolysis in *Nfatc1*-deficient CD8 + T cells. Like CD4 + T cells, CD8 + T cells also require c-Myc to undergo metabolic reprogramming and to engage in high rates of glycolysis upon TCR stimulation. During asymmetric division, CD8 + T cells generate daughter cells with distinct properties, such as different proteasome activities. Daughter cells with increased proteasome activity express memory CD8 + T cell markers, such as CD62L, IL-7R, and Tcf1. Pharmacologically activating proteasome activity reduces c-Myc expression and promotes memory CD8 + T cell differentiation [186]. TCR activation induces a gradient of IRF4 expression in a TCR affinity-dependent manner [187]. IRF4 is required for the expression of glycolysis-related genes, including those involved in glucose transportation (*Glut1* and *Glut3*) and glycolysis (*Hk2*, encoding hexokinase 2; *Aldoa*, *Aldoc*, and *Aldoart1*, encoding aldolases; and *Pfkfb3*, encoding 6-phosphofructo-2-kinase/fructose-2,6-biphosphatase 3). *Irf4* deficiency impairs glucose uptake, glycolysis, and CD8 + T cell survival [187]. Forced expression of FoxP3 in CD8 + T cells increases the expression levels of glycolysis-related genes, improves CD8 + T cell metabolic fitness, and promotes CD8 + T cell accumulation in tumors [188]. In CD4 + T cells, FoxP3 inhibits the expression of the glycolysis-related transcription factor c-Myc and suppresses glucose uptake and glycolysis [163]. These studies suggest that FoxP3 plays distinct roles under natural and forced expression conditions in CD4 + T cells and CD8 + T cells, respectively.

The protein von Hippel‒Lindau (VHL) was originally identified as a tumor suppressor [189]. Under normoxic conditions, the proline residue of HIF1α is hydroxylated. Then, HIF1α is ubiquitinated by VHL followed by proteasomal degradation [190]. Therefore, VHL is a negative regulator of HIF1α. Deletion of *Vhl* stabilizes HIF1α and promotes glycolysis and the antitumor response in CD8 + T cells [191]. Furthermore, *Vhl* deficiency confers tumor-infiltrating CD8 + T cells with tissue-resident memory T cell-like properties (such as high expression levels of CD103 and CD69) and elicits a better antitumor response [192].

mTOR activation promotes glycolysis. Inhibition of mTOR with rapamycin or RNA interference promoted the formation of memory CD8 + T cells in an LCMV infection model [193]. Tuberous sclerosis complex 1 (Tsc1) is expressed in naïve CD8 + T cells and suppresses the activation of mTORC1 (Fig. 2). *Tsc1* deficiency leads to excessive mTORC1 activity in CD8 + T cells [194]. *Tsc1* deficiency increases the glycolytic rate of CD8 + T cells. Although *Tsc1* deficiency does not fundamentally affect the formation of effector CD8 + T cell responses, the memory precursor effector cell population is affected by *Tsc1* deficiency. As a result, *Tsc1* deficiency impairs the formation of memory CD8 + T cells and the recall response to *Listeria monocytogenes* infection [195]. PTEN is known to be a tumor suppressor and a negative regulator of the PI3K pathway. Acylglycerol kinase (AGK) triggers the phosphorylation of PTEN, thereby inhibiting its phosphatase activity. *Agk* deficiency impairs CD8 + T cell glycolysis and antitumor T cell responses [196] (Fig. 2).

The IL-7/IL-7R signaling axis increases the cell surface expression of Glut1 and glucose uptake by T cells (Fig. 2). The tyrosine 449 residue of the IL-7R protein is required for activating the signal transducer and activator of transcription 5 (STAT5) and Akt pathways. Glucose uptake plays an important role in the pro-survival effects of IL-7 [197]. Deletion of *Il7r* in vivo, although it does not influence glucose uptake by T cells, reduces glycolytic rates, potentially by inhibiting the activities of glycolytic enzymes [198]. Notably, although IL-7-IL-7R signaling plays a crucial role in maintaining naïve T cell homeostasis, its activity requires tight regulation. Continuous IL-7 signaling causes naïve CD8 + T cell proliferation and IFN-γ-dependent cell death [199]. IL-2 increases the expression of c-Myc and promotes glycolysis in CD8 + T cells [51, 200]. Persistent IL-2 stimulation in CD8 + T cells leads to chronic activation of STAT5, which leads to the expression of tryptophan hydroxylase 1 [201]. Tryptophan hydroxylase 1 promotes the generation of 5-hydroxytryptophan from tryptophan. 5-Hydroxytryptophan stimulates the nuclear translocation of AhR. AhR, in turn, impairs antitumor T cell function by increasing the expression of inhibitory receptors and decreasing effector cytokine generation [201]. Moreover, IL-2-induced constitutively active STAT5 antagonizes the T cell exhaustion program driven by the transcription factor Tox. The IL-2-STAT5 pathway partially revives exhausted CD8 + T (Tex) cells and endows them with antitumor effects [202]. Therefore, the role of the IL-2-STAT5 pathway in regulating CD8 + T cell metabolism and function is context dependent.

T cell activation rapidly increases the expression of insulin receptors on T cells [203]. Insulin treatment increases the expression of Glut1 and promotes glucose uptake. Insulin receptor deficiency impairs antigen-driven T cell proliferation and inflammatory cytokine production. Furthermore, antiviral CD8 + T cells require insulin receptors to control infections by influenza virus [203]. Leptin increases glucose uptake by T cells and promotes T cell proliferation and the production of cytokines, such as IL-2 and IFNγ [204]. CD8 + T cells in tumors express higher levels of leptin receptor than CD8 + T cells in the spleen [205]. Oncolytic viruses engineered to express leptin promote the expression of T cell stemness-associated markers, such as IL-7R and Tcf1, in CD8+ TILs and enhance tumor control [205]. Fructose influences CD8 + T cell effector function in an extrinsic manner. A fructose-rich diet promotes mTOR activation and leptin production by adipocytes. Leptin, in turn, enhances antitumor CD8 + T cell responses [206].

Systemic bacterial infections induce acetate accumulation in plasma. Acetate increases the size of the pool of cellular acetyl-CoA, which is required for the acetylation of GAPDH. GAPDH acetylation increases GAPDH activity, promotes glycolysis, and enhances memory CD8 + T cell responses. Acetate pretreatment strengthens memory CD8 + T cell responses against bacterial infections [4]. The tumor microenvironment has low levels of glucose. Acetate is known to be an alternative carbon source for cancer cells. Acetate causes a change in the epigenetic landscape in glucose-restricted CD8 + T cells. As a result of this epigenetic reprogramming, *Ifng* gene transcription is enhanced. Reducing the expression levels of acetyl-CoA synthetase reverses acetate-induced IFN-γ production, suggesting that acetate induces epigenetic reprogramming in an acetyl-CoA synthetase-dependent manner [207].

CD8 + T cell activation is a signature of the Warburg effect in vitro. However, an elegant in vivo study using ^13^C-glucose infusion techniques revealed that CD8 + T cells activated under physiological conditions are more oxidative than CD8 + T cells activated in vitro. A ^13^C tracer labeling assay revealed that glucose is preferentially used to synthesize nucleotides and serine. These results highlighted the necessity of using in vivo approaches to study T cell metabolism under physiological conditions [208].

## Amino acid metabolism and CD4 + T cell activation and subset differentiation

Amino acid metabolism plays multifaceted roles in a wide spectrum of pathways that regulate T cell survival, activation, and function. The availability of amino acids is influenced by the development of diseases, such as cancer [209, 210]. In this section, we use several amino acids as examples to discuss the pathways through which amino acids regulate the activation and subset differentiation of CD4 + T cells and CD8 + T cells. We will also discuss how perturbations in amino acid availability influence T cell function and disease development.

### Glutamine

Although glutamine is defined as a nonessential amino acid, this amino acid is often added to cell media to achieve optimal cell growth. T cell activation increases the expression levels of genes involved in glutamine metabolism [3]. For example, T cell activation induces the expression of ASCT2 (encoded by *Slc1a5*), which mediates glutamine uptake and further leads to the activation of mTORC1 [211]. Glutamine deprivation or genetic deletion of c-Myc, a key transcription factor that plays a role in regulating T cell metabolic reprogramming, impairs the biosynthesis of polyamines and nucleotides in T cells [3]. T cells require glutamine to proliferate and secrete the effector cytokines IL-2 and IFNγ, but glutamine is not required for the expression of early activation markers, such as CD25, CD69, and CD98 [144].

**Fig. 3:**
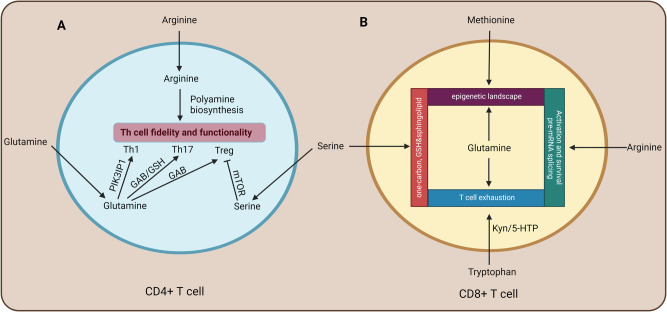
Amino acid metabolism modulates T cell survival and lineage commitment. **A** T cell activation increases the expression of amino acid transporters that import glutamine and arginine. Glutamine catabolism plays a crucial role in regulating the differentiation of naïve CD4 + T cells into Th1 cells, Th17 cells, or Treg cells. Excessive serine accumulation leads to mTOR hyperactivation and impairs the suppressive function of Treg cells. Arginine catabolism fuels polyamine biosynthesis and is necessary for maintaining CD4 + T cell subset identity. **B** The glutamine-glutamate-α-KG pathway shapes the epigenetic landscape, posttranslationally modifies proteins (i.e., glutarylation), and removes toxic ammonia in CD8 + T cells. CD8 + T cells require methionine and its downstream metabolite SAM to regulate histone methylation, STAT5 expression, and antitumor responses. Serine and glycine metabolism fuels one-carbon metabolism, glutathione generation, and de novo sphingolipid synthesis in CD8 + T cells. Tryptophan depletion or supplementation with its downstream product kynurenine inhibits T cell proliferation. Arginine metabolism promotes the activation and survival of antitumor CD8 + T cells. Arginine methylation in Sm proteins facilitates signal transduction by regulating pre-mRNA splicing of IL2rg and JAK3. α-KG α-ketoglutarate, GAB γ-aminobutyrate, GSH glutathione, Kyn kynurenine, 5-HTP 5-hydroxytryptophan. Images were created with Biorender.com, with permission

Glutaminolysis is not only required to support T cell proliferation but also modulates T cell subset differentiation (Fig. 3A). Glutaminase (encoded by *Gls*) is required for glutaminolysis. Glutamine deprivation promotes Treg cell polarization in vitro. Furthermore, Treg cells differentiated with glutamine deprivation exhibit greater proliferative potential and are more effective in restricting Teff cell responses [212]. Genetic deficiency of *Gls* or inhibition of glutaminase inhibits Th17 cell differentiation, decreases the expression of phosphoinositide-3-kinase-interacting protein 1 (encoded by *Pik3ip1*), and promotes Th1 cell lineage commitment [213]. Furthermore, after being transferred to tumor-bearing hosts, T cells preconditioned with glutamine restriction in vitro are better at controlling tumors than are those cultured without glutamine restriction [214]. These results appear to contradict previous findings showing that glutamine is required for T cell proliferation and cytokine production [144]. One possible explanation is that glutamine plays different roles in the early and late stages of T-cell activation. Complete inhibition of glutamine catabolism may impair the initial activation and proliferation of T cells. In the late stages, however, T cells switch to an alternative carbon source, glucose. This metabolic adaptation may account for the increase in cytokine production by T cells [213]. These results are reminiscent of those of another study that revealed that tumoricidal T cell function is enhanced following the inhibition of glycolysis [215]. Collectively, these studies indicate that nutrient restriction confers an advantage to T cells in terms of survival in the harsh metabolic tumor microenvironment.

Glutamate regulates Treg cell subset differentiation and their suppressive function. The catalytic subunit of glutamate cysteine ligase (encoded by the gene *Gclc*) catalyzes a rate-limiting step in glutathione synthesis from the substrates glutamate and cysteine [216]. *Gclc* deficiency increases the availability of serine, which leads to hyperactivation of mTOR and impairs the suppressive function of Treg cells (Fig. 3A). As a result, *Gclc* deficiency causes lethal autoimmune responses and enhances antitumor immunity [217]. Another study revealed that glutamate decarboxylase, an enzyme that converts glutamate to γ-aminobutyrate (GAB), is highly expressed in Treg cells and Th17 cells [218]. As a result, both Treg cells and Th17 cells have high amounts of GAB. Th17 cells express 4-aminobutyrate aminotransferase (ABAT) at higher levels than Treg cells. ABAT converts GAB to succinic semialdehyde and subsequently to succinate, which then fuels the TCA cycle and promotes Th17 cell differentiation. In Treg cells, due to low expression of ABAT, GAB is not catabolized inside the cells and is exported to the extracellular compartment. GAB then acts on the GABA receptor on the cell surface and promotes Treg cell differentiation in an autocrine manner [218] (Fig. 3A). ABAT deficiency in T cells ameliorates symptoms in the EAE mouse model.

### Arginine metabolism and CD4 + T cell differentiation

Arginine is a semiessential amino acid. Cells can synthesize arginine from citrulline but still need to import arginine from extracellular compartments when arginine is needed in large amounts [219–221]. Arginine is depleted in tumor tissues. Both tumor cells and myeloid cells in tumors express arginase 1 and hydrolyze arginine [222–229]. Arginine is either imported into cells through the cationic amino acid transporter CAT2B and hydrolyzed by intracellular arginase 1 [230] or directly hydrolyzed by extracellular arginase 1 in the extracellular compartment [231, 232]. Arginine is broken down into urea and ornithine [219], which are precursors of the synthesis of putrescine, spermine, and spermidine [219].

T cell proliferation is inhibited when cells are cultured in arginine-free medium, suggesting that arginine plays a key role in supporting T cell proliferation [233–235]. Arginine plays multifaceted roles in regulating T cell activation. Arginine is required for surface expression of the CD3ζ chain [235, 236]. Arginine restriction inhibits TCR signaling [237]. Notably, glutamine, glycine, leucine, or lysine depletion does not change CD3ζ chain expression, suggesting that arginine plays a unique role in TCR signal transduction [235]. Arginine promotes the expression of the cell cycle-related protein cyclin D3 [238, 239]. Arginine depletion inhibits the dephosphorylation of the actin-binding protein cofilin, thereby impairing immunological synapse formation and T cell activation [240–242]. Arginine is also indispensable for IL-2 signaling because arginase deprivation suppresses the expression of the IL-2 receptor α [235, 236]. Furthermore, arginase deprivation inhibits the expression of multiple cytokines, such as IFNγ and TNFα [235, 242].

CD4 + T cell subset differentiation is regulated by arginine metabolism (Fig. 3A). Arginine and the downstream polyamine synthetic pathway are predicted to be closely associated with Th17 pathogenicity according to an algorithm known as Compass. This prediction is supported by experimental evidence suggesting that pharmacological and genetic perturbation of polyamine metabolism promotes Treg cell differentiation at the expense of Th17 cells [243]. A major substrate for synthesizing polyamines is arginine. Th1 cells, Th2 cells, Th17 cells, and Treg cells undergo active polyamine synthesis. Th1 cells and Th2 cells consume arginine for polyamine synthesis at much higher rates than Th17 cells and Treg cells, suggesting that Th17 cells and Treg cells use different substrates for polyamine synthesis from Th1 cells and Th2 cells [244]. Arginase 1 is induced in CD4 + T cells in the lung during influenza infection. Arginase 1 restrains Th1 cell-mediated viral clearance and lung pathology [245]. Therefore, arginine metabolism and subsequent polyamine biosynthesis play pivotal roles in regulating CD4 + T cell differentiation in disease settings.

## Regulation of CD8 + T cell-mediated immune responses by amino acid metabolism

### Glutamine

Glutamine is deaminated to form glutamate and α-ketoglutarate (α-KG), which supports anaplerosis of the TCA cycle and plays a crucial role in regulating histone methylation and chromatin accessibility (Fig. 3B). Glutamine and α-KG promote the IL-2-induced effector cell gene program [246]. α-KG is required for the activity of several enzymes, such as ten-eleven translocation methylcytosine dioxygenases, HIF prolyl 4-hydroxylases, and histone lysine demethylases. Glutarate inhibits ten-eleven translocation of methylcytosine dioxygenase 2, HIF prolyl 4-hydroxylase 1, and histone lysine demethylase 4 C/6 A. This inhibition of α-KG-dependent enzymes by glutamate is consistent with its role in the epigenetic regulation of CD8 + T cells, such as histone methylation and DNA demethylation [247]. Glutamate has been used by CD8 + T cells as a substrate to modify proteins posttranslationally via a process known as glutarylation [247]. Glutylation has also been reported in other cell types [248, 249]. The pyruvate dehydrogenase complex promotes the transport of pyruvate into mitochondria and its subsequent conversion to acetyl-CoA [111–114]. The catalytic enzyme unit of the pyruvate dehydrogenase complex, dihydrolipoamide acetyltransferase (PDHE2), is glutarylated in CD8 + T cells [247]. This posttranslational modification affects lipoic acid conjugation to PDHE2 lysine residues, a process that is required for the activity of PDHE2 [250]. Ammonia, a metabolic byproduct of amino acid deamination, is toxic and induces cell death [251, 252]. α-KG serves as an ammonia scavenger to sustain antiviral CD8+ cell responses [253]. Mechanistically, the malate shuttle enzyme Got1 catalyzes an atypical chemical reaction to generate α-KG and aspartate from oxaloacetate and glutamate. α-KG assimilates and detoxifies ammonia, thereby helping maintain the metabolic fitness of Tex cells [253]. A recent study showed that memory CD8 + T cells use the urea and citrulline cycles to remove ammonia, which is crucial for memory CD8 + T cell formation and longevity [254]. These studies suggest that CD8 + T cells use more than one strategy to detoxify ammonia. Overall, the glutamine-glutamate-α-KG metabolic pathway plays a key role in regulating T cell bioenergetics, the epigenetic landscape, and ammonia metabolism.

### Arginine

When arginine is limited, the expression of argininosuccinate synthetase 1 increases, and arginine is synthesized from citrulline in tumor cells. In T cells, however, arginine restriction causes repressive histone methylation and globally compact chromatin and results in low expression of argininosuccinate synthetase 1. As a result, T cells do not engage in this argininosuccinate synthetase 1-mediated synthesis of arginine. Arginine starvation hinders T cell activation [233]. Intracellular arginine is rapidly consumed and metabolized after TCR activation. Supplementing arginine increases the rate of oxidative respiration at the expense of glycolysis. CD8 + T cells treated with arginine have a greater survival capacity and display features of central memory T cells. Adoptive transfer of these arginine-conditioned CD8 + T cells inhibited tumor growth to a greater extent than the transfer of unconditioned CD8 + T cells [255].

In addition to its role in cellular metabolism, arginine methylation also regulates the development of CD8 + T cells and other cell types. The arginine methyltransferase PRMT5 is expressed at high levels in T cells. Symmetric dimethylation of Sm proteins at arginine residues requires PRMT5. Sm proteins are ribonucleoproteins that promote the splicing of *Il2rg* and *Jak3* pre-mRNAs. Therefore, PRMT5 is indispensable for the appropriate expression of γc and JAK3 and for signal transduction downstream of γc family cytokines in CD8 + T cells and other immune cell types [256] (Fig. 3B).

### Methionine

Methionine is an essential amino acid. Methionine is converted to S-adenosyl-methionine (SAM) by the enzyme methionine adenosyltransferase II alpha. SAM is a methyl group donor for the methylation of DNA and proteins [257]. Tumor-initiating cells depend on methionine to maintain their tumor-initiating capability [258]. Tumor cells express high levels of the methionine transporter large neutral amino acid transporter small subunit (encoded by *SLC43A2*), which competes for methionine with CD8+ TILs and disrupts methionine metabolism in CD8+ TILs (Fig. 3B). A reduction in methionine and SAM in T cells causes the loss of H3K79me2, downregulates the expression of STAT5, and impairs the antitumor immunity of CD8 + T cells [259]. Furthermore, the inhibition of SAM production in tumor cells by deleting methionine adenosyltransferase II alpha ameliorates the CD8 + T cell exhaustion phenotype and delays tumor growth [260]. Therefore, targeting tumor methionine metabolism could be a promising strategy to antagonize T cell exhaustion in the tumor microenvironment.

### Serine

T cells use serine as a substrate for multiple metabolic pathways, such as one-carbon metabolism, the folate cycle, nucleotide synthesis, glutathione generation, and de novo sphingolipid synthesis (Fig. 3B). T cells obtain serine through either transport from the extracellular compartment or de novo synthesis.

Phosphoglycerate dehydrogenase (PHGDH) mediates the use of glycolysis-derived carbons for serine biosynthesis [261]. *Phgdh* deficiency reduced the diverted carbon flow from glycolysis to serine biosynthesis and impaired CD8 + T cell expansion in a mouse model of *Listeria monocytogenes* infection [208]. In addition to its role in driving serine biosynthesis, PHGDH has an enzyme activity-independent function [262].

The removal of extracellular serine using a serine-restricted diet inhibits the antibacterial response of CD8 + T cells [263]. During bacterial infections, CD8 + T cells increase the expression levels of genes related to serine and glycine synthesis, one-carbon metabolism, the folate cycle, and nucleotide synthesis [263]. This finding is reminiscent of similar findings made in CD4 + T cells. TCR activation in CD4 + T cells triggers strong one-carbon metabolic features. Serine and glycine contribute to the biosynthesis of nucleotides, thereby sustaining T cell growth and proliferation [264]. Using the tracer deuterium (D)-labeled serine, an elegant study showed that one-carbon units are mainly generated in mitochondria in CD4 + T cells [264]. Glycine is a product of one-carbon metabolism. Aged T cells have smaller mitochondria and lower respiratory capacity than young T cells. Glycine promotes the activation of naïve T cells from aged mice [265].

Serine is also required for glutamate-cysteine ligase-mediated synthesis of glutathione. Glutathione, a neutralizer of ROS, is required for T cell metabolic fitness and TCR activation-induced metabolic reprogramming. A deficiency in *Gclc* (encoding a glutamate cysteine ligase) affects major metabolic regulators, such as mTOR, NFAT, and c-Myc. As a result, *Gclc* deficiency ameliorates T-cell-mediated autoimmune disorders [266]. Although the main focus of this review is T cells, experimental results obtained from other cell types, such as macrophages, also suggest that the serine-glutathione synthesis pathway plays a key role in regulating inflammatory responses. For example, glutathione is required for optimal IL-1β mRNA expression, which is induced by lipopolysaccharide. Inhibition of serine synthesis ameliorates lipopolysaccharide-induced IL-1β transcription and sepsis [267].

Furthermore, serine is also a substrate for de novo sphingolipid synthesis. Our laboratory has demonstrated that de novo sphingolipid synthesis is crucial for maintaining the metabolic fitness of antiviral CD8 + T cells. Deficiency of *Sptlc2*, an enzyme that catalyzes the rate-limiting step of de novo sphingolipid synthesis (i.e., condensing serine and palmitoyl coenzyme A into 3-keto-sphinganine), causes prolonged activation of mTORC1 and ER stress-induced cell death, thereby impairing the survival and proliferation of antiviral CD8 + T cells [1]. In addition to sphingolipid synthesis, acid sphingomyelinase-mediated breakdown of sphingolipid is required for CD8 + T-cell-dependent clearance of LCMV infection in mice [268].

### Tryptophan

T cells do not synthesize tryptophan or obtain tryptophan from the diet. The majority of free tryptophan is metabolized via the kynurenine pathway [269]. The enzymes tryptophan-2,3-dioxygenase or indoleamine 2,3-dioxygenase 1 and 2 (IDO1 and IDO2) catalyze the degradation of tryptophan to N-formylkynurenine, which is a key step of the kynurenine pathway, followed by subsequent conversion of N-formylkynurenine to kynurenine, 3-hydroxykynurenine, quinolinic acid and picolinic acid [269]. Tumor cells and myeloid cells in tumors express high levels of IDO1 [269–275], resulting in a low abundance of tryptophan [270] and high levels of kynurenine [270, 276].

Tryptophan depletion or supplementation with kynurenine or picolinic acid inhibits T cell proliferation [227, 271, 274, 277] (Fig. 3B). Tryptophan deprivation impairs T cell proliferation by affecting TCR proximal signaling. This finding is supported by the following experimental evidence. Tryptophan depletion inhibits anti-CD3- or concanavalin A-induced T cell proliferation [277]. However, PMA- and ionomycin-induced T cell proliferation and cytokine production are not markedly affected [271, 274, 278]. When amino acids are limited, uncharged tRNAs accumulate and activate the general control nonderepressible 2 (GCN2) kinase and eventually trigger the integrated stress response [279]. Certain studies have suggested that tryptophan deprivation activates GCN2 kinase in CD4 + T cells [227] and that IDO-expressing DCs activate GCN2 kinase in CD8 + T cells [280]. However, there are also reports showing that GCN2 kinase activation is not detected in tumor-infiltrating T cells [281]. These observations suggest that tryptophan deprivation induces an integrated stress response in a context-dependent manner.

The exact pathways through which T cell proliferation is inhibited by IDO in vivo are not fully understood. The traditional view is that IDO reduces the availability of an essential amino acid, thereby suppressing T cell responses. However, the concentrations of tryptophan in tumors are above the threshold for triggering an integrated stress response [281]. Therefore, the lack of nutrients itself may not account for the immunosuppressive function of IDO in vivo [281, 282]. An alternative explanation is that tryptophan-derived metabolites contribute to IDO-mediated T cell inhibition. Kynurenine accumulates in the TME and drives the exhaustion of tumor-infiltrating T cells by activating AhR [283]. Moreover, kynurenine competes with other amino acids, such as methionine and leucine, that are transported by Slc7a5 to T cells, thereby impairing T cell-mediated immune responses [284]. Furthermore, IL-2 induces the expression of tryptophan hydroxylase 1 in CD8+ TILs, thus facilitating the conversion of tryptophan to 5-hydroxytryptophan (5-HTP). Further analysis suggested that 5-HTP promotes CD8 + T cell exhaustion by activating AhR [181]. Therefore, the contribution of tryptophan depletion to the formation of an immunosuppressive TME is likely dependent on downstream metabolic products.

## Concluding remarks

In this review, we discuss recent research progress on how lipids, glucose, and amino acids regulate CD4^+^ and CD8^+^ T cell activation, subset differentiation, and effector function. Increasing evidence suggests that these metabolites not only provide energy and building blocks to support cell growth and survival but also regulate T cell intracellular signal transduction and the epigenetic landscape to influence cell differentiation and effector function. A clear trend in this field is to combine biochemical and biophysical approaches to dissect the molecular mechanisms through which these metabolites regulate T cell subset differentiation and function in physiological and pathological settings.
